# Accuracy of Lower Extremity Alignment Correction Using Patient-Specific Cutting Guides and Anatomically Contoured Plates

**DOI:** 10.3390/jpm15070289

**Published:** 2025-07-04

**Authors:** Julia Matthias, S Robert Rozbruch, Austin T. Fragomen, Anil S. Ranawat, Taylor J. Reif

**Affiliations:** 1 Orthopedic Surgery—Limb Lengthening and Complex Reconstruction Service, Hospital for Special Surgery, Weill Cornell Medicine, New York, NY 10021, USA; julia.matthias@cuanschutz.edu (J.M.); rozbruchsr@hss.edu (S.R.R.); fragomena@hss.edu (A.T.F.); 2 Orthopedic Surgery—Sports Medicine Service, Hospital for Special Surgery, Weill Cornell Medicine, New York, NY 10021, USA; ranawatanil@hss.edu

**Keywords:** cutting guide, 3D printing, patient-specific instrumentation, distal femur osteotomy, high tibial osteotomy

## Abstract

**Background/Objectives**: Limb malalignment disrupts physiological joint forces and predisposes individuals to the development of osteoarthritis. Surgical interventions such as distal femur or high tibial osteotomy aim to restore mechanical balance on weight-bearing joints, thereby reducing long-term morbidity. Accurate alignment is crucial since it cannot be adjusted after stabilization with plates and screws. Recent advances in personalized medicine offer the opportunity to tailor surgical corrections to each patient’s unique anatomy and biomechanical profile. This study evaluates the benefits of 3D planning and patient-specific cutting guides over traditional 2D planning with standard implants for alignment correction procedures. **Methods**: We assessed limb alignment parameters pre- and postoperatively in patients with varus and valgus lower limb malalignment undergoing acute realignment surgery. The cohort included 23 opening-wedge high tibial osteotomies and 28 opening-wedge distal femur osteotomies. We compared the accuracy of postoperative alignment parameters between patients undergoing traditional 2D preoperative X-ray planning and those using 3D reconstructions of CT data. Outcome measures included mechanical axis deviation and tibiofemoral angles. **Results**: 3D reconstructions of computerized tomography data and patient-specific cutting guides significantly reduced the variation in postoperative limb alignment parameters relative to preoperative goals. In contrast, traditional 2D planning with standard non-custom implants resulted in higher deviations from the targeted alignment. **Conclusions**: Utilizing 3D CT reconstructions and patient-specific cutting guides enhances the accuracy of postoperative limb realignment compared to traditional 2D X-ray planning with standard non-custom implants. Patient-specific instrumentation and personalized approaches represent a key step toward precision orthopedic surgery, tailoring correction strategies to individual patient anatomy and potentially improving long-term joint health. This improvement may reduce the morbidity associated with lower limb malalignment and delay the onset of osteoarthritis. **Level of Evidence**: Therapeutic Level III.

## 1. Introduction

Lower limb malalignment, including varus and valgus deformities, alters the mechanical load distribution across the knee and predisposes individuals to the development of osteoarthritis. Surgical correction through procedures such as distal femoral osteotomy (DFO) and high tibial osteotomy (HTO) aims to restore mechanical alignment, redistribute joint-loading forces, and delay disease progression [1,2]. These procedures are commonly performed to offload an arthritic medial or lateral compartment of the knee, but can also be used prophylactically to correct limb deformities such as genu varum (“bowleg”) or genu valgum (“knock-knee”) and to optimize joint biomechanics prior to the onset of degeneration. The accuracy of the intended correction is paramount, as both undercorrection and overcorrection can lead to persistent supraphysiologic articular forces and premature progression of osteoarthritis.

Planning the correct osteotomy has historically been done on 2D radiographs using trigonometry, and the execution in the operating room relies heavily on the measured short limb of an isosceles triangle [3]. However, reliance on 2D imaging introduces the potential for error due to limited spatial information, particularly in complex multiplanar deformities. In contrast, advances in 3D imaging, computer-assisted design, and manufacturing technologies have introduced the use of patient-specific instrumentation (PSI) and cutting guides, which are tailored to the unique anatomical structure of a patient.

Today, computerized tomography is readily available at most hospitals, and with advances in medical imaging software, three-dimensional (3D) bone models can now be generated preoperatively [4]. Prior to surgery, these models can undergo virtual osteotomy to plan precise mechanical alignment correction. Furthermore, 3D printing technologies enable the creation of patient-specific cutting guides and anatomically contoured plates, offering a more tailored and reproducible approach to realignment surgery.

These personalized tools represent a significant advance in the field of personalized or precision medicine. By integrating patient-specific anatomical data into surgical planning, the surgical approach can be tailored to the unique geometry of the patient’s limb, enabling highly individualized corrections. This approach has the potential to improve surgical accuracy, enhance long-term outcomes, and reduce complications associated with malalignment. Despite these important implications, there remains limited evidence comparing the real-world performance of 3D-planned, patient-specific cutting guides to conventional 2D preoperative planning and standard implants in osteotomy procedures.

The objective of this study was to compare the accuracy of limb alignment correction in patients undergoing DFO and HTO surgeries using advanced 3D CT-based planning versus conventional 2D radiographic planning and techniques. The primary outcome measures were the absolute error and accuracy in the actual correction versus the planned correction, as measured by mechanical axis deviation and mechanical tibiofemoral angle. We hypothesize that the use of 3D reconstructions and patient-specific instrumentation would result in more precise achievement of preoperative alignment goals, reducing the variability and risk of malcorrection. By addressing this question, our work contributes to the broader field of personalized medicine, highlighting how individualized preoperative planning and surgical execution can optimize musculoskeletal outcomes and reduce morbidity associated with malalignment.

## 2. Materials and Methods

### 2.1. Study Design and Setting

We conducted a retrospective analysis of patients who underwent acute realignment surgery for varus and valgus lower limb malalignment with opening-wedge high tibial osteotomy or distal femur osteotomy at a single institution from 2020 to 2022. Following institutional review board approval, patient charts were reviewed for demographic information (age and sex) and surgical procedures performed. We included patients in the study who underwent either traditional 2D preoperative X-ray planning with standard non-custom implants or 3D reconstructions of CT data with patient-specific cutting guides and plates. 2D alignment and corrections were measured and planned via Sectra picture archiving and communication system (PACS) software (Sectra AB IDS7 version 25.2, Linkoping, Sweden). CT data of the hip, knee, and ankle along with standing radiographs were submitted to Bodycad (Quebec City, QC, Canada) for 3D bone reconstruction, virtual osteotomy correction, and fabrication of patient-specific cutting guides and plates. Subjects were excluded if weight-bearing radiographs of the entire limb were not available to assess mechanical alignment either pre- or postoperatively. The demographics and surgical information of the patients are summarized in Table 1.

### 2.2. Surgical Correction

Surgeries were performed by fellowship-trained surgeons specializing in osteotomy correction of lower limb malalignment. The surgical approaches were the same for 2D and 3D corrections. The distal femur was approached laterally by incising the iliotibial band and elevating the vastus lateralis. The tibia was approached medially by elevating the pes anserine tendons and subperiosteal elevation of the superficial medial collateral ligament. 2D osteotomies were performed with a small oscillating saw over a wire. 3D osteotomies were performed with an oscillating saw through a patient-specific cutting box. Lamina spreaders were used to facilitate the opening-wedge corrections. The 2D corrections were stabilized with Tomofix plates and locking screws (Depuy Synthes, Warsaw, IN, USA). The 3D corrections were stabilized with personalized Bodycad plates and locking screws. Closure was performed with standard suture repair of the IT band or pes tendons depending on the approach and subcuticular closure of the skin.

### 2.3. Radiographic Analysis

We assessed limb alignment parameters pre- and postoperatively in patients with varus and valgus lower limb malalignment undergoing acute realignment surgery. The primary outcome measurements were mechanical axis deviation (MAD) and tibiofemoral angles (mTFA). These parameters were evaluated using weight-bearing X-rays of the entire lower limb in anteroposterior projection, both preoperatively and postoperatively.

#### The Alignment Parameters Assessed Were

Mechanical Tibiofemoral Angle (mTFA): This is the angle between the mechanical axis of the femur (a line connecting the center of the femoral head to the center of the intercondylar fossa) and the mechanical axis of the tibia (a line connecting the point between the intercondylar eminences to the center of the talus).

Mechanical Axis Deviation (MAD): This is the distance between the center of the knee (the point between the tibial intercondylar eminences) and the line connecting the center of the femoral head to the center of the talus.

### 2.4. Data Analysis

The primary outcome measures were the error and accuracy of the correction of the mTFA and MAD, defined as follows:

Error Assessment: Postsurgical error was quantified by calculating the difference between the goal and the postoperative values of MAD or mTFA.

Postoperative Accuracy Assessment: Postsurgical accuracy was assessed by calculating the relative error, which was determined by dividing the postsurgical error by the planned correction (the difference between the preoperative measurement and the goal). Accuracy was then defined as 1 minus the relative error.

Statistical analysis was performed using GraphPad Prism Version 10.5.0 (673). Radiographic and demographic numeric data were compared using unpaired Student’s *t*-tests when data met the assumptions of normality and equal variances, and the Mann–Whitney U test when normality was not met. Variance differences were assessed using the F-test for equality of variances. A *p*-value of less than 0.05 was considered statistically significant for all assessments.

## 3. Results

A total of 19 patients undergoing distal femoral osteotomy (DFO) and 19 patients undergoing high tibial osteotomy (HTO) were included in the study. Among the DFO patients, nine underwent conventional 2D preoperative planning with standard non-custom implants (2D group), and ten patients received 3D preoperative planning with CT imaging and customized cutting guides (3D group). For HTO, nine patients received conventional 2D planning, and ten underwent 3D planning. Contralateral legs treated at later time points were counted separately, resulting in 28 DFO cases (13 with 2D planning and 15 with 3D planning) and 23 HTO cases (12 with 2D planning and 11 with 3D planning).

### 3.1. MAD and mTFA Error Analysis

We first evaluated the mechanical axis deviation (MAD) and mechanical tibiofemoral angle (mTFA) errors in DFO and HTO patients by comparing the postoperative alignment to the preoperatively defined goals. Analyses were conducted separately for DFO and HTO patients, as well as in combination.

**Figure 1:**
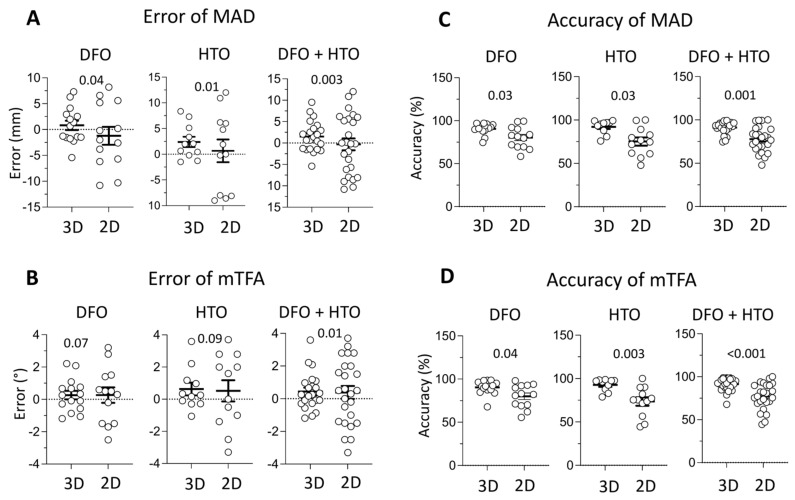
Postsurgical lower limb alignment error and accuracy. Postoperative error (**A**,**B**) and accuracy (**C**,**D**) in patients undergoing distal femoral osteotomy (DFO), high tibial osteotomy (HTO), and both procedures combined, comparing conventional 2D preoperative planning with standard non-custom implants (2D) versus CT-guided preoperative planning with customized cutting guides (3D). *Error* (MAD in mm or mTFA in °): Calculated as the postoperative MAD or mTFA minus the preoperative goal; *Accuracy* (MAD or mTFA in %): (1 − Error/[Preoperative measurement − Goal]) × 100. The graphs display means with standard deviation (SD). Statistical significance was assessed using an F-test for equality of variances in (**A**,**B**). For (**C**,**D**), an unpaired Student’s *t*-test was used when assumptions of equal variance and normality were met; otherwise, the Mann–Whitney U test was applied. A *p*-value < 0.05 was considered statistically significant.

As shown in Figure 1A, while the mean MAD in both the 2D and 3D planning groups was close to the surgical goal, the data variance was significantly smaller in the 3D planning group (Figure 1A MAD error, DFO: 3D 0.8 ± 3.46, 2D −1.22 ± 6.25, *p* = 0.038; HTO: 3D 2.4 ± 3.33, 2D −1.26 ± 6.71, *p* = 0.035; DFO/HTO: 3D 1.5 ± 3.52, 2D −1.24 ± 6.34, *p* = 0.0083).

Similarly, Figure 1B demonstrates a trend toward a lower mTFA variance in the 3D group, though this difference did not reach statistical significance when DFO and HTO were analyzed separately. However, when combined, the reduction in variance reached statistical significance (Figure 1B mTFA error, DFO: 3D 0.26 ± 1.03, 2D 0.26 ± 1.73, *p* = 0.065; HTO: 3D 0.63 ± 1.31, 2D −0.23 ± 2.19, *p* = 0.118; DFO/HTO: 3D 0.44 ± 1.13, 2D 0.03 ± 1.94, *p* = 0.015).

### 3.2. Accuracy Measurements

To further validate these findings, we assessed the alignment accuracy, defined as 1 minus the ratio of the absolute error (MAD or mTFA) to the planned preoperative correction magnitude. Again, the DFO and HTO patients were analyzed separately and together.

As shown in Figure 1C,D, the 3D planning group achieved significantly higher accuracy in both the DFO and HTO procedures, consistently exceeding 90%. In contrast, patients receiving conventional 2D preoperative planning and standard non-custom implants achieved lower accuracy, ranging from 76% to 80% in both the DFO and HTO groups. These differences were statistically significant across both separate and combined analyses (Figure 1C MAD accuracy, DFO: 3D 90.85 ± 6.25, 2D 80.35 ± 12.73, *p* = 0.0105; HTO: 3D 92.22 ± 8.08, 2D 77.05 ± 15.72, *p* = 0.0122; DFO/HTO: 3D 91.24 ± 6.93, 2D 78.76 ± 14.04, *p* = 0.0002. Figure 1D mTFA accuracy, DFO: 3D 90.34 ± 7.88, 2D 80.08 ± 13.4, *p* = 0.0186; HTO: 3D 92.76 ± 7.17, 2D 76.93 ± 16.18, *p* = 0.0103; DFO/HTO: 3D 91.31 ± 7.55, 2D 78.64 ± 14.49, *p* = 0.0003).

## 4. Discussion

The primary aim of this study was to compare the accuracy and error of distal femoral osteotomy (DFO) and high tibial osteotomy (HTO) surgeries using 3D and 2D planning techniques. Our findings demonstrate that when surgical groups were combined, the 3D technique resulted in significantly lower correction error and improved overall accuracy.

Several factors contribute to the limitations of traditional 2D preoperative planning. One key challenge is the difficulty of accurately reproducing the planned opening-wedge triangle intraoperatively. The size and shape of the wedge is dependent on the start point, trajectory, and depth of the osteotomy, and even small deviations can lead to significant angular discrepancies [5]. To mitigate this, the correction can be checked with an intraoperative alignment rod, but this is dependent on the rotation of the leg and does not represent the weight-bearing position of the leg utilized during planning [6]. Thus, even a seemingly perfect intraoperative correction may not translate to proper alignment on postoperative standing radiographs [7]. Finally, non-contoured “off the shelf” plates can deform the bone during screw placement, especially while placing cortical screws to oppose the plate to the bone [8]. This mechanical distortion can further compromise the intended correction, underscoring the technical challenges of 2D-guided procedures.

Using 3D bone model planning and patient-specific cutting guides allows for greater control over the start point location and trajectory of the osteotomy. The guides are personalized to the unique contours, bumps, and ridges of each patient’s bone to obtain an ideal fit. However, attention still needs to be paid to correct placement of cutting guides, since soft tissue impingement and bending of the plastic guides can still introduce errors, and guides can still be placed in subtly incorrect positions despite patient-specific contouring [9]. While CT imaging is usually obtained in the supine position, the resulting bone models can be matched to the preoperative standing radiograph to approximate the weight-bearing configuration prior to manipulation. Nevertheless, ligamentous laxity following correction remains difficult to quantify and remains an estimation.

Patient-specific anatomically contoured plates offer additional advantages by resisting deformation of the corrected bone during screw placement. Since the direct apposition of the plate to the bone is accounted for in the 3D planning, they permit the use of locking screws without altering the correction, ensuring that the planned alignment is preserved during fixation. In combination, these improvements contribute to more accurate corrections, a finding supported by the results of this study.

Our findings align with several recent studies that also found the accuracy benefits of patient-specific surgical instrumentation. Fayard et al. noted a significantly higher rate of achieving target alignment in high tibial osteotomies when using patient-specific cutting guides (90%) compared to standard freehand techniques (65%) [10]. Similarly, Jacquet et al. demonstrated a significantly increased accuracy in distal femur opening-wedge osteotomy (0.43 vs. 3.95 degrees) using patient-specific guides [11], along with a shorter operative time and fewer fluoroscopy images. However, the decrease in radiation exposure intraoperatively is very likely outweighed by the increased exposure associated with the preoperative CT scan [12].

A systematic review by Dasari et al. of 14 studies utilizing medial opening-wedge HTO using patient-specific instrumentation found an overall error of 0.5 degrees and a significantly reduced mechanical axis error in four comparative studies [13], findings that are consistent with the results of the present study. Similarly, a systematic review by Aman et al. that included both opening- and closing-wedge HTOs and DFOs found that five of six comparative studies demonstrated higher accuracy using patient-specific instrumentation techniques [14].

Importantly, the improved accuracy observed with 3D planning may have meaningful clinical implications. Even small residual malalignments can increase joint loading, accelerate cartilage wear, and raise the risk of early osteoarthritis progression. Sharma et al. observed that minimal varus alignment of the knee of 178° or less was significantly associated with greater odds of medial osteoarthritis progression, with an adjusted OR of 1.29 per 1° varus [15]. By more closely matching the preoperative alignment goals, 3D techniques may help delay the onset of secondary osteoarthritis, reduce the need for early joint replacement, and improve long-term patient-reported outcomes such as pain relief, function, and satisfaction. Ultimately, long-term studies of conversion to knee arthroplasty using 2D versus 3D correction techniques will determine the impact of improved accuracy.

Incorporating these patient-specific techniques into surgical practice also introduces important resource considerations. While 3D planning initially requires additional steps, such as CT scans, image processing, and the manufacturing of custom guides, improved surgical accuracy may reduce revision surgeries, complication rates, and shorten operative times. While Thomas et al. reported that PSI-guided TKA patients had statistically significantly lower mean total hospital costs and reduced readmission rates compared to those without PSI [16], thereby reducing the economic burden on the healthcare system, Beyer et al. found that cost savings due to reduced numbers of instrument trays and time in the operating room did not offset the additional costs associated with PSI technology [17]. These contrasting findings highlight the need for a formal cost-effectiveness analysis to comprehensively assess the economic value of patient-specific instrumentation and cutting guides in routine orthopedic practice, including not only direct surgical costs but also long-term outcomes, including revision rates, functional recovery, and patient satisfaction. Improved accuracy, however, may be associated with better long-term outcomes, including a reduction in complications such as over- or undercorrection that may necessitate re-operation. Notably, varus and valgus malalignment have been associated with a 3.6- to 4.9-fold increased risk of progression to knee osteoarthritis [15], emphasizing the importance of achieving precise alignment. Knee osteoarthritis represents a major economic burden, affecting over 32.5 million adults in the United States and contributing to an estimated USD 185.5 billion in annual healthcare costs [18]. More than 80% of this burden is attributable to knee OA, particularly among adults aged 45 and older, underscoring its disproportionate impact [19]. These considerations highlight the importance of prevention and surgical precision in realignment procedures, which may ultimately outweigh the immediate costs associated with PSI.

Importantly, the integration of 3D planning and patient-specific instrumentation aligns with the current shift towards personalized medicine in orthopedics. These patient-centered techniques enable tailored corrections based on each patient’s unique anatomy, alignment, and biomechanical needs, rather than applying a uniform surgical strategy. This approach recognizes the heterogeneity of deformities and leverages precision tools to deliver individualized care, potentially optimizing both immediate surgical outcomes and long-term joint health.

This study has limitations. First, the sample size was relatively small and included both distal femur and proximal tibia osteotomies, introducing heterogeneity into the analysis. Further research in a larger patient cohort is needed to better assess the accuracy advantages of the 3D technique by specific osteotomy type. Second, alignment measurements were obtained from a single standing radiograph with the patella facing forward, potentially introducing errors due to unaccounted rotational differences. Third, the non-randomized design of this study introduces potential sources of bias, including selection bias, as treatment groups were not randomly assigned and may reflect underlying differences in patient characteristics or surgeon decision-making. The retrospective nature of the study also introduces the possibility of reverse causation, limiting our ability to infer causal relationships. As highlighted by Karamitros et al., it is critical to distinguish correlation from causation in surgical research and to recognize the inherent limitations of retrospective observational studies [20]. Furthermore, our data were collected from a single center, which should be considered when assessing the generalizability of our findings. While our results are consistent with those reported by other groups, a larger multicenter, randomized controlled trial would be necessary to strengthen the external validity of our conclusions. In addition, alignment measurements were performed by a single observer, which may have introduced measurement bias.

We acknowledge that the inclusion of both valgus and varus correction by lateral femur and medial tibia opening-wedge techniques introduces heterogeneity into our analysis. However, this heterogeneity also reflects the real-world diversity of clinical presentations and may enhance the external validity of our findings. Despite this heterogeneity, we observed a consistent and statistically significant improvement in alignment accuracy with 3D preoperative planning and patient-specific cutting guides compared to conventional 2D radiographic planning. While our study focused exclusively on opening-wedge techniques, further research is warranted to determine whether these findings extend to closing-wedge osteotomies.

We also acknowledge that the surgeons performing the 3D procedures were different from those performing the 2D-guided procedures. However, the surgeons performing the 2D procedures had greater osteotomy experience, so the improved accuracy of the 3D technique cannot be due to experiential volume. While this variation may have introduced technical discrepancies, it may also increase the external validity of the results by demonstrating that the improved accuracy is achievable across different surgical teams with variable experience in the procedure.

Lastly, although our data demonstrate a clear radiographic improvement in postsurgical alignment accuracy with 3D planning, further research is needed to explore whether the enhanced accuracy of 3D planning translates into measurable long-term benefits for patients, including improved functional, patient-reported, and joint preservation outcomes.

## 5. Conclusions

In conclusion, our results demonstrate that the mechanical axis correction error and overall accuracy were significantly improved using 3D planning and patient-specific instrumentation compared to conventional 2D techniques in both opening-wedge HTO and DFO procedures. These findings highlight the clinical importance of precision in alignment correction, as even small improvements in surgical accuracy may help delay the onset of secondary osteoarthritis, reduce the need for early joint replacement, and improve long-term functional outcomes and patient safety.

Beyond technical improvements, adopting patient-specific technologies represents a meaningful step forward in personalized orthopedic care. Ultimately, integrating precision tools into surgical patient-centered planning and execution has the potential to advance personalized medicine in orthopedics, offering patients tailored solutions that improve clinical outcomes.

## Figures and Tables

**Table 1:** Participant demographic summary for patients undergoing distal femoral osteotomy (**A**) or high tibial osteotomy (**B**). The 2D and 3D cohorts demonstrated no significant differences in age.

**A**	***Distal Femoral Osteotomy***	**2D Planning**	**3D Planning**	***p*-Value**
	Age at surgery (±SD)	34.18 (±8.33)	33.93 (±16.46)	0.66
	% female patients (% female femurs)	67 (69)	70 (67)	
	N patients (N femurs)	9 (13)	10 (15)	
**B**	***High tibial osteotomy***	**2D planning**	**3D planning**	***p*-value**
	Age at surgery (±SD)	44.67 (±16.52)	38 (±10.49)	0.29
	% female patients (% female tibiae)	22 (16.7)	30 (36.4)	
	N patients (N tibiae)	9 (12)	10 (11)	

## Data Availability

The raw data supporting the conclusions of this article will be made available by the authors on request.

## Abbreviations

The following abbreviations are used in this manuscript:

DFO	Distal femoral osteotomy
HTO	High tibial osteotomy
MAD	Mechanical axis deviation
mTFA	Mechanical tibiofemoral angle
